# The MDM2-p53 pathway is involved in preconditioning-induced neuronal tolerance to ischemia

**DOI:** 10.1038/s41598-018-19921-x

**Published:** 2018-01-25

**Authors:** Rebeca Vecino, Maria C. Burguete, Teresa Jover-Mengual, Jesus Agulla, Verónica Bobo-Jiménez, Juan B. Salom, Angeles Almeida, Maria Delgado-Esteban

**Affiliations:** 1 Institute of Biomedical Research of Salamanca, University Hospital of Salamanca, University of Salamanca, Calle Zacarías González 2, 37007 Salamanca, Spain; 2 Institute of Functional Biology and Genomics, University of Salamanca, CSIC, Calle Zacarías González 2, 37007 Salamanca, Spain; 3 Unidad Mixta de Investigación Cerebrovascular (UMIC), Instituto de Investigación Sanitaria La Fe, and Departamento de Fisiología, Universidad de Valencia, Hospital Universitario y Politécnico La Fe, Av. Fernando Abril Martorell, 106, 46026 Valencia, Spain; 4 Stroke Program. Department of Neurology. Hospital Clínico Universitario, Valladolid. IESCYL Av. Ramón y Cajal, 3, 47003 Valladolid, Spain

## Abstract

Brain preconditioning (PC) refers to a state of transient tolerance against a lethal insult that can be evoked by a prior mild event. It is thought that PC may induce different pathways responsible for neuroprotection, which may involve the attenuation of cell damage pathways, including the apoptotic cell death. In this context, p53 is a stress sensor that accumulates during brain ischemia leading to neuronal death. The murine double minute 2 gene (MDM2), a p53-specific E3 ubiquitin ligase, is the main cellular antagonist of p53, mediating its degradation by the proteasome. Here, we study the role of MDM2-p53 pathway on PC-induced neuroprotection both in cultured neurons (*in vitro)* and rat brain (*in vivo)*. Our results show that PC increased neuronal MDM2 protein levels, which prevented ischemia-induced p53 stabilization and neuronal death. Indeed, PC attenuated ischemia-induced activation of the p53/PUMA/caspase-3 signaling pathway. Pharmacological inhibition of MDM2-p53 interaction in neurons abrogated PC-induced neuroprotection against ischemia. Finally, the relevance of the MDM2-p53 pathway was confirmed in rat brain using a PC model *in vivo*. These findings demonstrate the key role of the MDM2-p53 pathway in PC-induced neuroprotection against a subsequent ischemic insult and poses MDM2 as an essential target in ischemic tolerance.

## Introduction

Ischemic tolerance (IT) is a well-known phenomenon in which brief non-injurious preconditioning stimulus (preconditioning, PC; i.e exposure to low doses of N-methyl-D-aspartate, NMDA-PC) confer robust neuroprotection against a subsequent severe ischemic damage^1–8^. The endogenous mechanisms activated during experimental PC could provide an effective experimental tool leading to understand how the brain protects itself. Therefore, experimental PC would allow us to develop new therapeutic strategies against ischemic brain damage. Although the molecular mechanisms underlying IT are not yet fully clarified, the attenuation of the apoptotic cell death may be involved^9–11^.

The protein p53 is a stress sensor that accumulates during brain ischemia leading to neuronal death. Several evidences from both animal and human studies demonstrated that cerebral ischemia initiates a cascade of metabolic events that involve the stabilization and activation of p53^12–16^. Additionally, the genetic ablation or pharmacological inhibition of p53, blocked neuronal apoptosis after ischemia in both *in vitro* and *in vivo* models^17–19^. Recently, several studies revealed that p53 stabilization and the subsequent nuclear translocation of p53 lead to the transcriptional enhancement of numerous genes, such as p21, PUMA and p53^20,21^, which contributes to caspase-mediated neuronal apoptosis^22^. The main regulator of p53 levels is the E3 ubiquitin ligase murine double minute 2 (MDM2), which can be modulated by different stimulus, including hypoxia, oncogene activation and DNA damage, which in turns control p53 stabilization^23–25^. In fact, p53 binding to MDM2 is required for its degradation by the proteasome preventing the transcriptional activation of p53 regulated genes^26–29^. Cellular stress causes modifications in both p53 and MDM2 proteins, which decreases the avidity of p53 for MDM2^23^. Thus, the activation of kinases by DNA damage or ischemia-induced metabolic alterations^23^ promotes p53 phosphorylation of key region-binding sites for MDM2^23,26^. Under phisiological conditions, MDM2 and p53 form an auto-regulatory feedback loop which acts as a repressor of p53 activity in the cell^24,27,30^. Under this loop, p53 stimulates the expression of MDM2, which, in turn, promotes p53 degradation^27,31^. Here, we examined the MDM2-p53 signaling pathway on PC-induced IT in neurons. Our results showed that PC increased MDM2 protein levels, which prevented ischemia-induced p53 stabilization. Furthermore, PC attenuated ischemia-induced activation of the p53/PUMA/caspase-3 signaling pathway and promoted neuronal survival against a subsequent ischemic damage. Disruption of the MDM2-p53 interaction with nutlin-3a treatment abrogated PC-induced neuroprotection. Finally, the relevance of the MDM2-p53 pathway was confirmed in the rat brain using a validated PC *in vivo* model. PC *in vivo* increased MDM2 protein levels, induced p53 destabilization and reduced cerebral infarction after ischemia. Then, our findings demonstrate the key role of the MDM2-p53 signaling pathway in neuroprotection induced by PC against a subsequent ischemic insult and poses MDM2 as an essential target in IT.

## Results

### NMDA-PC prevents ischemia-induced p53 stabilization and neuronal apoptosis

**Figure 1:**
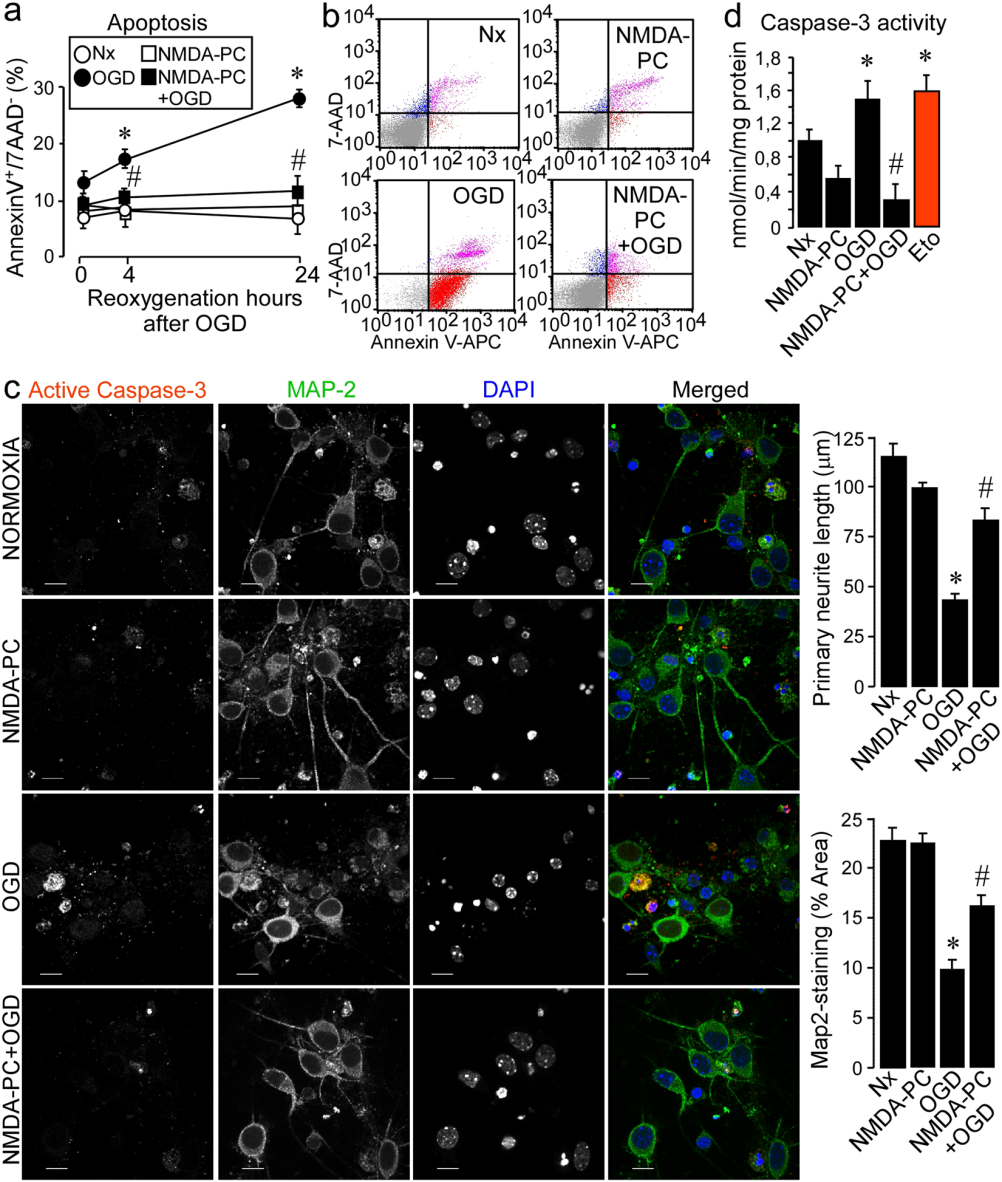
NMDA-PC prevents ischemia-induced neuronal apoptotic death. Mouse cortical neurons (9–10 DIV) were exposed to a validated *in vitro* model of NMDA-PC and neuronal apoptosis (**a**,**b**) was analyzed by flow cytometry. Annexin V-APC stained cells that were 7AAD negative were considered to be apoptotic (AnnexinV+/7AAD−). (**a**) OGD induced neuronal apoptosis in a time-dependent manner, which was prevented by NMDA-PC. (**b**) Flow cytometry plots showed that NMDA-PC prevented OGD-induced neuronal apoptosis, as shown by the decrease in the percentage of apoptotic neurons (lower right, red), in comparison to OGD condition. (**c**) Immunofluorescence images, the primary neurites length and Map-2-staining area quantification revealed that NMDA-PC prevented neurite degeneration caused by ODG. Scale bar: 10μm. (**d**) NMDA-PC prevented the activation of caspase-3 induced at 4 hours after OGD, as revealed by both (**c**) immunostaining and (**d**) fluorimetry (Neurons treated with apoptosis inductor, 10 μM etoposide for 24 hours were treated as control of apoptosis). Data are means ± S.E.M. (n = 5 independent neuronal cultures). Statistical analysis of the results was evaluated by one-way analysis of variance, followed by the least significant difference multiple range test. Student’s t-test was used for comparisons between two groups of values. In all cases, p < 0.05 was considered significant. *p < 0.05 *versus* Nx. ^#^p < 0.05 *versus* OGD. Relative percentages of neurons with active caspase-3-staining/Map-2-staining are presented in Supplementary Fig. S1.

First, neurons were exposed to a validated *in vitro* model of PC^4^ (Table 1) and we tested whether NMDA-PC (20 µM NMDA, 2 hours) protected neurons from a severe ischemic insult (oxygen and glucose deprivation; OGD, 90 min). As shown in Fig. 1a,b, OGD time-dependently induced neuronal apoptosis, which was prevented by NMDA-PC, as revealed by flow cytometry analysis. Accordingly, NMDA-PC also prevented neurite degeneration (Fig. 1c,e), the activation of caspase-3 induced by OGD, as revealed by both fluorimetry assay (Fig. 1d) and immunostaning (Fig. 1e) and neuronal necrosis and cell damage at 4 hours after OGD, which were measured by trypan blue staining (see supplementary Fig. S4b) and LDH release (Fig. S4c), respectively. These results validates the NMDA-PC method utilized and confirm that preconditioned neurons displayed neuroprotection against ischemia.

**Table 1:** Experimental NMDA-PC model of neurons in primary culture. Mouse cortical neurons at 9–10 DIV were exposed to four different conditions: I) a group of cells (Normoxia; Nx group) was incubated at 37 °C in a humidified atmosphere of 95% air/5% CO_2_ in buffered Hanks’ solution. Under these condition, oxygen concentrations in the incubation medium were 190 ± 15 μM as measured with a Clark-type oxygen electrode; II) a second group of cells was exposed to a moderated concentration 20 μM NMDA for 2 hours NMDA-preconditioning; NMDA-PC); III) group of cells exposed to oxygen and glucose deprivation for 90 min (OGD) or IV) 20 μM NMDA for 2 hours prior to a subsequent lethal oxygen glucose deprivation (OGD; 90 min) (NMDA-PC + OGD). Neurons were then incubated in cultured medium for further 0, 4 or 24 hours of reoxygenation.

Group of cells	Treatment and time periods		
	NMDA-PC for 2 h	OGD for 90 min	Reoxygenation for 0, 4 or 24 hours
1) Nx	Medium change	Medium change	Medium change
2) NMDA-PC	**20** μ**M NMDA**	Medium change	Medium change
3) OGD	Medium change	**OGD**	Medium change
4) NMDA-PC + OGD	**20** μ**M NMDA**	**OGD**	Medium change

**Figure 2:**
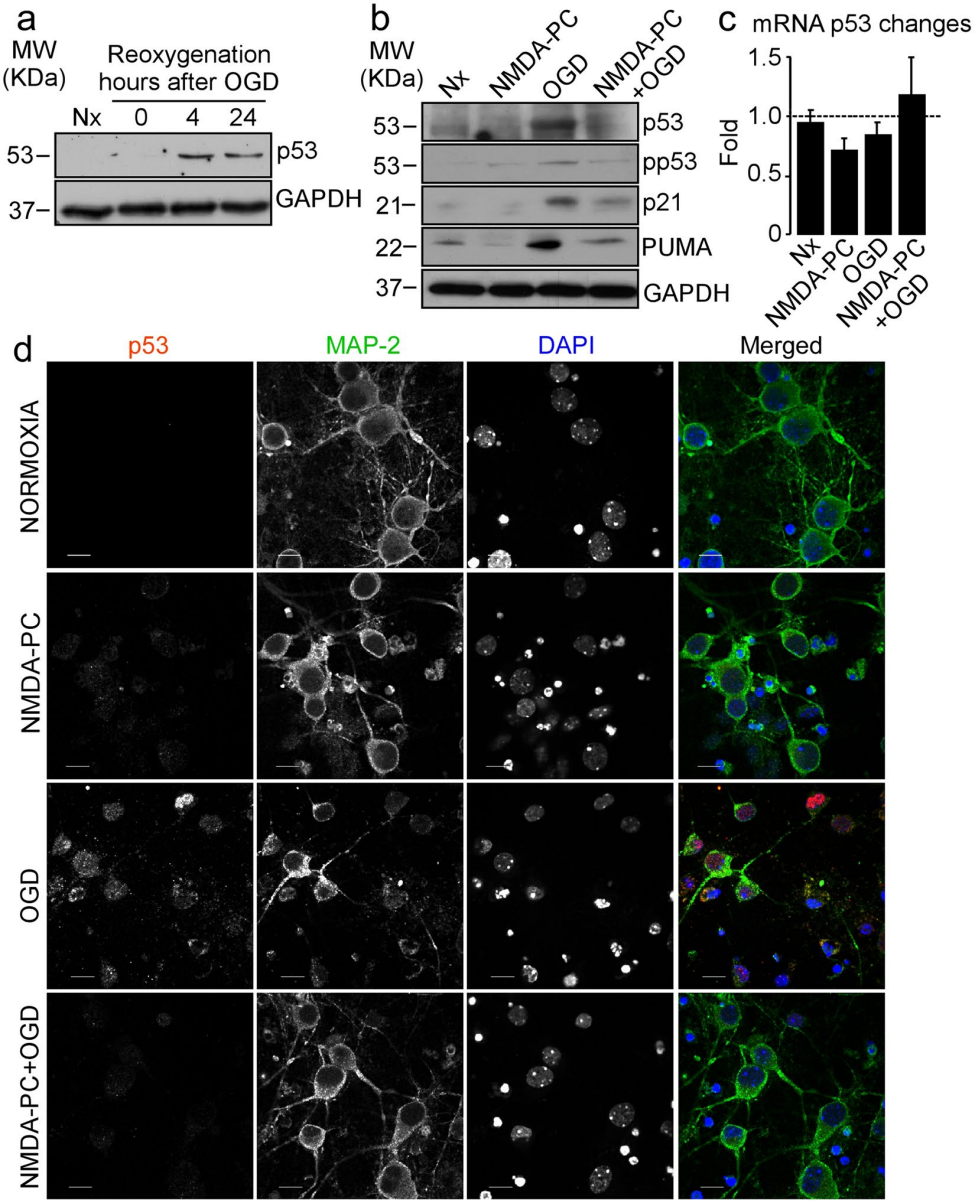
NMDA-PC promotes p53 destabilization throught a posttranslational mechanism. Mouse cortical neurons from fetal *wild type* (wt) or p53-null mice (*knockou*t, ko) (9–10 DIV) were exposed to a validated *in vitro* model of NMDA-PC (Table 1). (**a**) OGD induced p53 stabilization at 4 hours after OGD. (**b**) OGD induced the accumulation of phosphorylated p53 form (pp53, Ser 15) and its targets p21 and PUMA as revealed by western blotting. GADPH protein levels were used as loading control. A representative western blot is shown out of four. (**c**) RT-qPCR analysis of p53 gene reveals that p53 mRNA remained unaltered after OGD. (**d**) Fluorescence microphotographs after immunostaining for p53 (in red) and Map-2 (in green) confirmed that NMDA-PC prevented the accumulation of p53 induced by OGD in neurons. Scale bar: 10 μm. Relative percentages of neurons with p53-staining/Map-2-staining are presented in Supplementary Fig. S1. Relative protein abundances quantification Fig. 2a and Fig. 2b are presented in Supplementary Fig. S2 and that “full-length blots/gels are presented in Supplementary Fig. S3.

**Figure 3:**
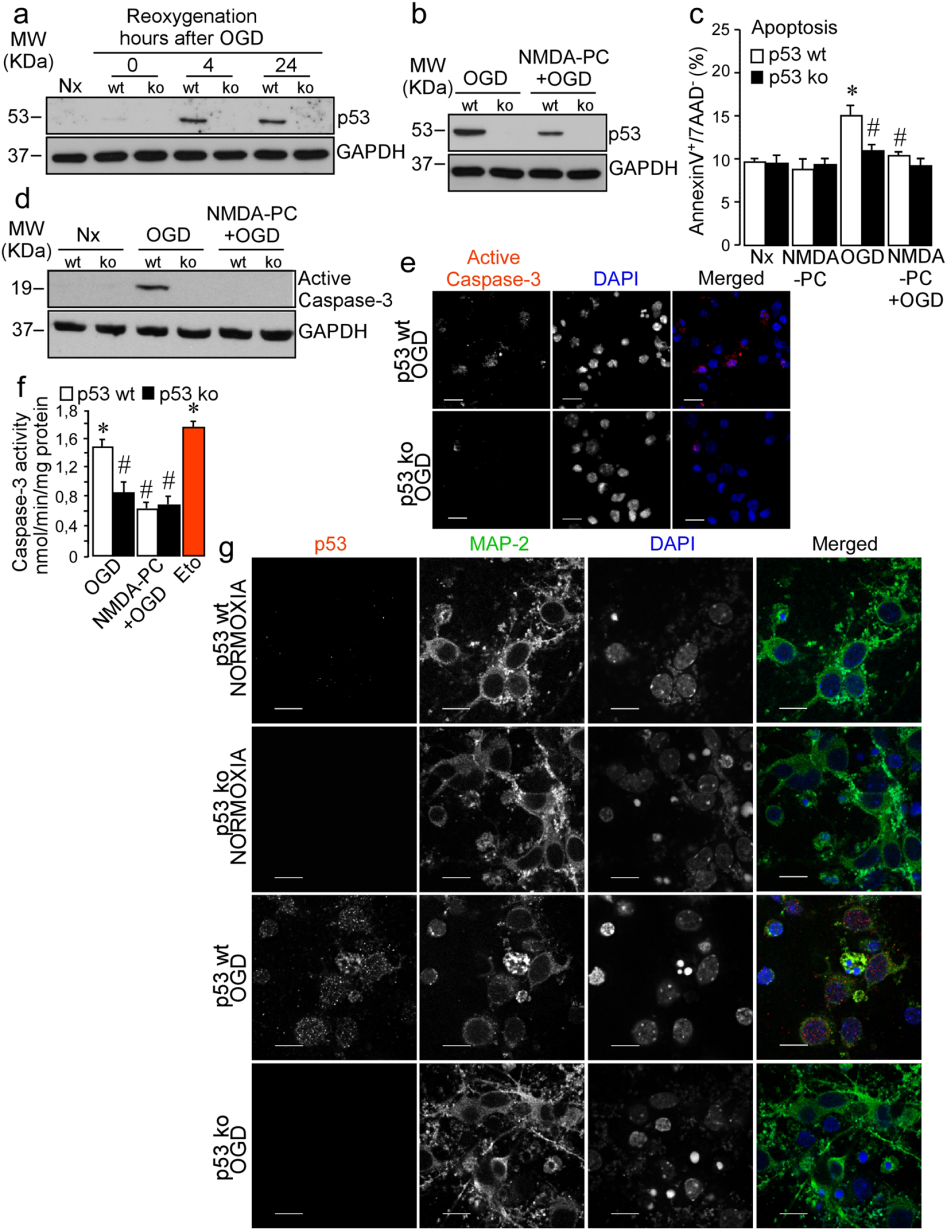
NMDA-PC prevents ischemia-induced neuronal apoptosis pathway mediated by p53. Cortical neurons from p53 wt or p53 ko mouse were exposed to a validated *in vitro* model of NMDA-PC (Table 1) and neuronal extracts were analyzed by western blotting. (**a**) At 4 hours after OGD, p53 stabilization was observed in wt, but not in p53 ko, neurons, (**b**) which was prevented by NMDA-PC. The lack of p53 totally prevented apoptosis caused by OGD at 4 hours after the ischemic insult, as revealed by (**c**) flow cytometry and active caspase-3 analyzed by (**d**) western blot, (**e**) immunofluorescence and (**f**) fluorimetry analysis. (**c**) The percentage of annexin V-APC stained neurons that were 7AAD negative were considered to be apoptotic (AnnexinV+/7AAD−). GADPH protein levels were used as loading control. (**e**) Fluorescence microphotographs of both wt and ko neurons after immunostaining for active-Caspase-3 (red). Scale bar: 20 μm. (**g**) immunostaining for p53 (red) and Map-2 (green). Scale bar: 15 μm. Data are means ± S.E.M. (n = 3 independent neuronal cultures). Statistical analysis of the results was evaluated by one-way analysis of variance, followed by the least significant difference multiple range test. Student’s t-test was used for comparisons between two groups of values. In all cases, p < 0.05 was considered significant. *p < 0.05 *versus* Nx. ^#^p < 0.05 *versus* OGD. Relative percentages of neurons with protein-staining are presented in Supplementary Fig. S1. Relative protein abundances quantification Fig. 3a, Fig. 3b,d are presented in Supplementary Fig. S2, the “full-length blots/gels are presented in Supplementary Fig. S3 and that Nx controls are shown in Fig. S4d.

The transcription factor p53 is an important regulator of apoptosis and cellular stress responses. Therefore, to examine the possible involvement of p53 in NMDA-PC-induced neuroprotection, next we measured p53 expression in neurons under conditions described in Table 1. Accordingly with our previous results^32^, western blot analysis revealed that reoxygenation following OGD promoted p53 stabilization in a time-dependent manner (Fig. 2a). However, NMDA-PC prevented the accumulation of p53 (Fig. 2b), as well as its transcriptional targets p21 and PUMA, induced by reoxygenation (4 hours) after OGD. In contrast, p53 mRNA remained unaltered (Fig. 2c). Furthermore, NMDA-PC prevented the accumulation of phosphorylated p53 (pp53 Ser15) caused by OGD (Fig. 2b). These results indicates that NMDA-PC abrogates ischemia-induced p53 stabilization by a posttranslational (phosphorylation) mechanism. Finally, co-immunostaining with anti-Map2 and anti-p53 confirm that NMDA-PC prevented p53-accumulation in neurons induced by the ischemic insult (Fig. 2d). All these results demonstrate that NMDA-PC prevents the accumulation of p53, which may be involved in neuroprotection. Next, we used cortical neurons from p53 knock-out (ko) mice to demonstrate whether the NMDA-PC-mediated neuroprotection by preventing ischemia-induced p53 pathway activation. First, we confirm that OGD induced p53 stabilization in wild type (wt), but not in p53 KO, neurons, which was prevented by NMDA-PC (Fig. 3a,b). We further found that genetic deletion of p53 totally prevented apoptosis caused by OGD at 4 hours after the insult, as revealed by flow cytometry analysis (Fig. 3c), western blot (Fig. 3d) and immunofluorescence (Fig. 3e; see Nx control in supplementary Fig. S4d) and fluorimetry (Fig. 3f). Then, the prevention of p53 stabilization after ischemia, which occurs after NMDA-PC (Figs 2b and 3b), is neuroprotective. All these results demonstrate that NMDA-PC confers neuroprotection against ischemia by promoting p53 destabilization.

### NMDA-PC regulates the MDM2-p53 pathway to promote neuroprotection

**Figure 4:**
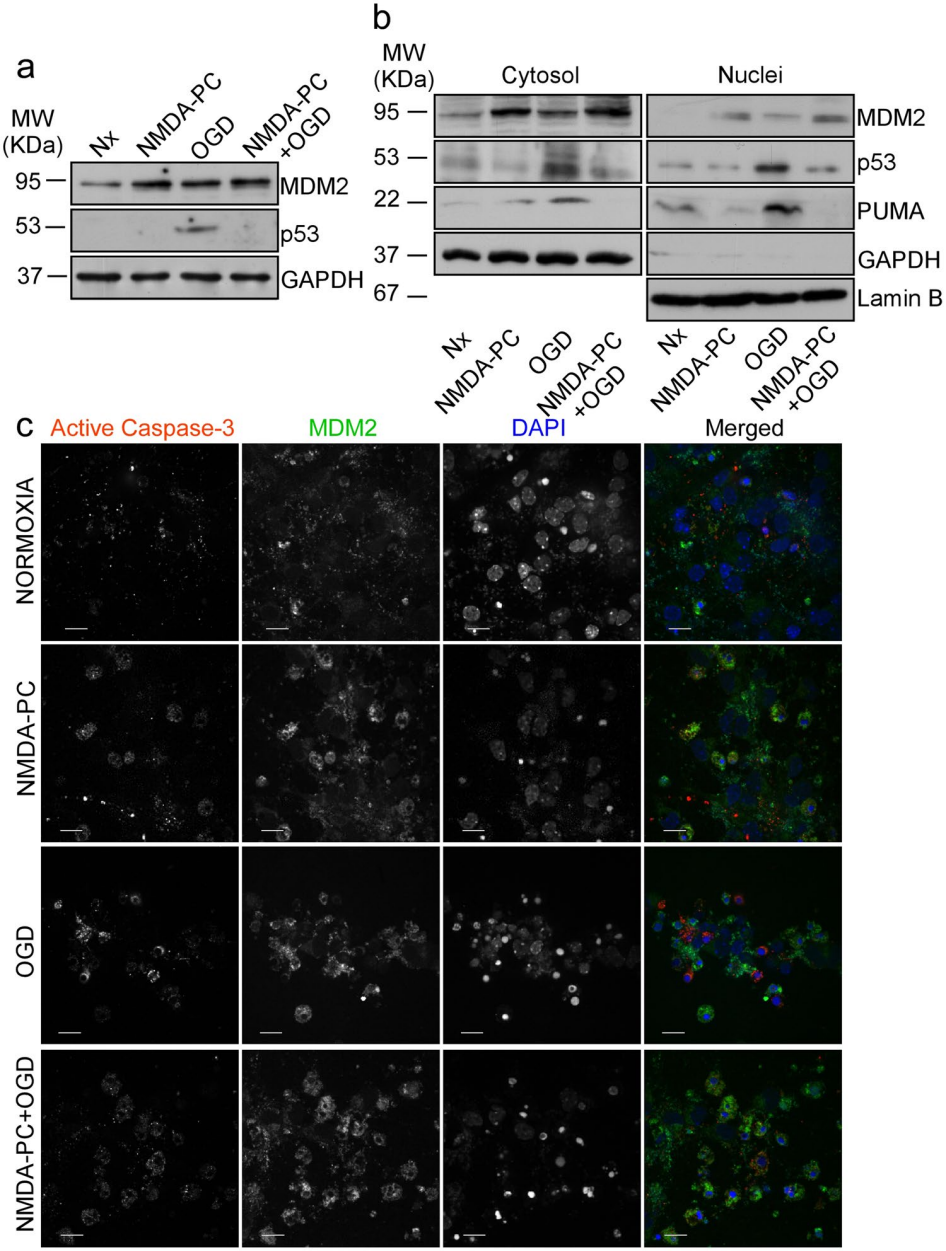
NMDA-PC increases MDM2 protein levels and promotes p53 nuclear and cytosolic destabilization. Mouse cortical neurons (9–10 DIV) were exposed to a validated *in vitro* model of NMDA-PC (Table 1). Levels of the E3-ubiquitin ligase MDM2 and p53 were detected by western blotting. GADPH protein levels were used as loading control. A representative western blot image is shown out of three. (**a**) NMDA-PC increased MDM2 levels at 4 hours after OGD. (**b**) This effect occurred in both nuclei and cytosol, together with a decrease in levels of p53 and its target PUMA. Lamin B and GAPDH protein levels were used as nuclear and cytosolic loading control, respectively. (**c**) Confocal images showed that NMDA-PC increased MDM2 levels expression (green) after OGD, which preferentially located in the nucleus (DAPI; blue), and prevented OGD-induced activation of caspase-3 (in red). Scale bar: 20 μm. Relative percentages of neurons with active Caspase-3/MDM2-staining are presented in Supplementary Fig. S1. Relative protein abundances quantification Fig. 4a and Fig. 4b are presented in Supplementary Fig. S2 and that “full-length blots/gels are presented in Supplementary Fig. S3.

**Figure 5:**
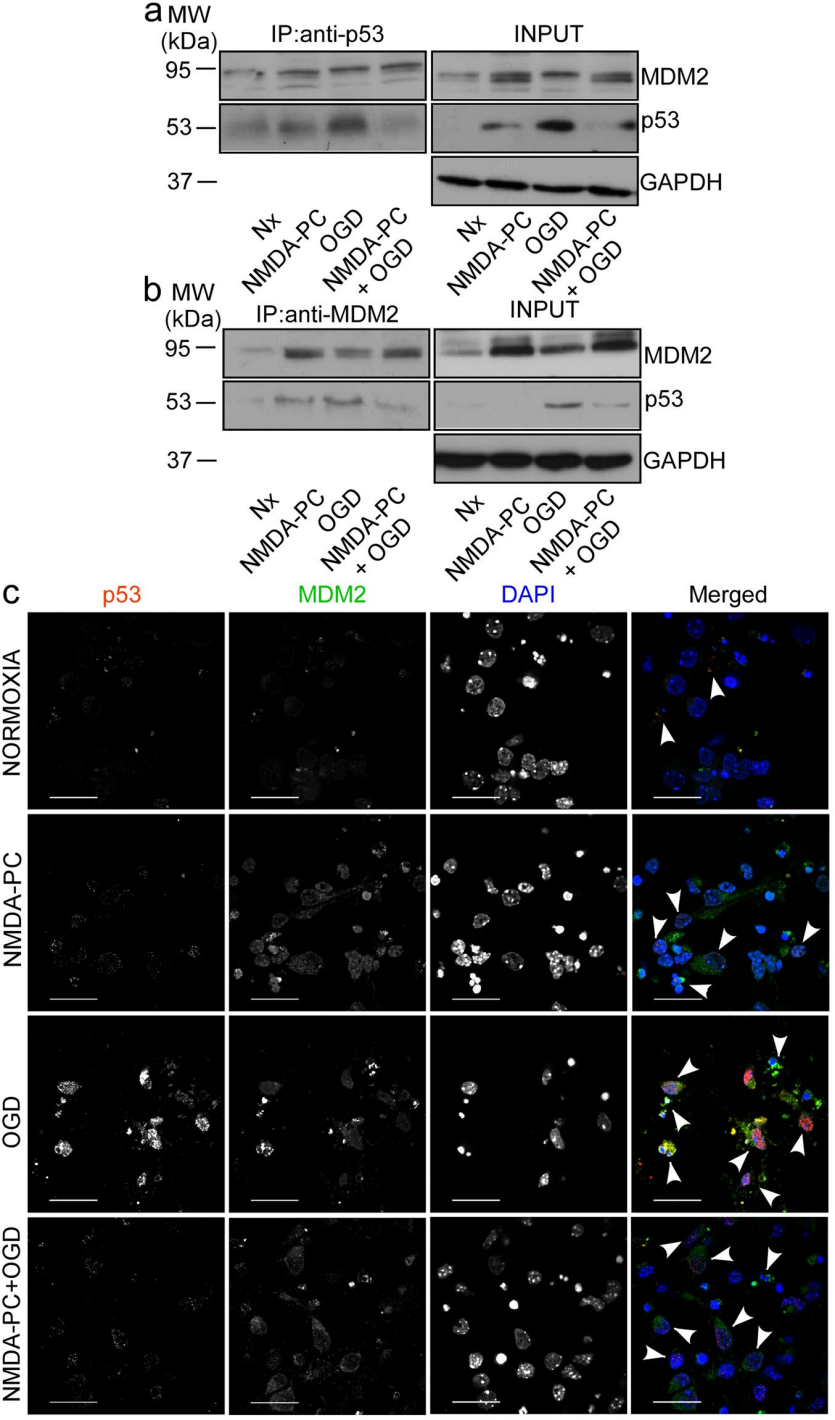
NMDA-PC promotes MDM2-p53 interaction, which prevents ischemia-induced p53 stabilization. Mouse cortical neurons (9–10 DIV) were exposed to a validated *in vitro* model of NMDA-PC (Table 1). Neuronal extracts were obtained and immunoprecipitated with anti-p53 (**a**) and anti-MDM2 (**b**) antibodies. Of the whole cellular extracts used for immunoprecipitation, 10% were loaded on SDS-PAGE as an input control. A representative western blot image is shown of three. (**a**,**b**) Co-immunoprecipitation assays revealed that p53 co-precipitated with MDM2. (**a**,**b**) NMDA-PC prior OGD, promotes MDM2-p53 interaction. (**c**) Fluorescence microphotographs revealed that NMDA-PC increases MDM2 protein levels, which appears to be the main effect for NMDA-PC promoting MDM2-p53 interaction at 4 hours after OGD. Although OGD promoted MDM2-p53 interaction, this effect was not enough to prevent OGD-induced p53 stabilization.White arrowheads show MDM2-p53 interaction. Scale bar = 20 μm. Relative percentages of neurons with p53-staining/MDM2-staining are presented in Supplementary Fig. S1. Relative protein abundances quantification Fig. 5a and Fig. 5b are presented in Supplementary Fig. S2 and that “full-length blots/gels are presented in Supplementary Fig. S3.

Given our data indicating that NMDA-PC-promoted neuroprotection is mediated by p53 destabilization, we next focused on the master regulator of p53, MDM2. Our results showed that NMDA-PC increased MDM2 protein level expression and prevented ischemia-induced p53 stabilization (Fig. 4a). It is known that MDM2 binding to p53 is necessary for p53 degradation by the proteasome^27,33^. Moreover, it has been identificated that specifically, MDM2 promotes p53 cytosolic destabilization^23,34^. Accordingly, next we investigated whether NMDA-PC-increased MDM2 protein levels affected p53 localization. Thus, nuclei and cytosol neuronal fractions were obtained separately and protein levels were analyzed by western blotting. As shown in Fig. 4b, NMDA-PC increased MDM2 levels at 4 hours after OGD in both nuclei and cytosol, together with a decrease in p53 levels and its transcriptional target PUMA. Moreover, confocal images showed that NMDA-PC increased MDM2 levels expression after OGD and prevented OGD-induced activation of caspase-3 (Fig. 4c). Next, we studied the effect of NMDA-PC on MDM2-p53 interaction. Co-immunoprecipitation assays using anti-p53 (Fig. 5a) and anti-MDM2 (Fig. 5b) antibodies revealed that p53 coprecipitated with MDM2 (Fig. 5a and b). Our results showed that NMDA-PC increases MDM2 protein levels, which appears to be the main effect for NMDA-PC promoting MDM2-p53 interaction at 4 hours after OGD, as revealed by confocal imaging (Fig. 5c). Although OGD promoted MDM2-p53 interaction, this effect was not enough to prevent p53/MDM2 relative protein abundance induced by OGD, as revealed by western blot analyses (Fig. 5a,b) and immunofluorescence (Fig. 5c). All these results indicates that NMDA-PC increases MDM2 protein level expression, which promotes its interaction with p53 prior OGD, leading p53 nuclear and cytosolic destabilization and preventing OGD-induced p53-mediated neuronal apoptotic death.

**Figure 6:**
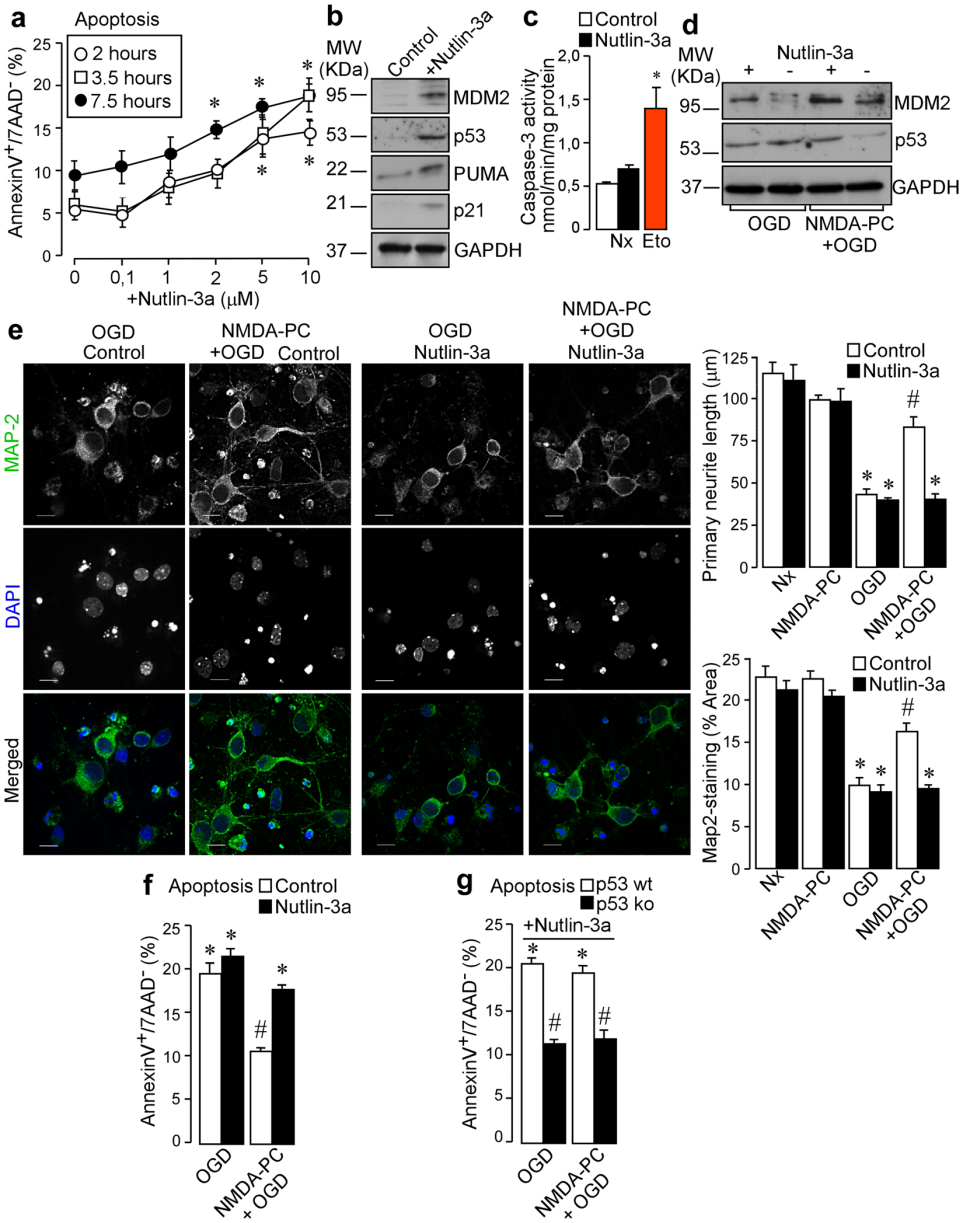
Pharmacological disruption of MDM2-p53 interaction abrogates NMDA-PC-caused neuroprotection after ischemia. Neurons (9–10 DIV) were treated with different concentrations of nutlin-3a (0–10 μM), a potent an specific inhibitor of MDM2 activity, at time periods indicated. (**a**) Nutlin-3a induced a dose-time dependent apoptotic effect in neurons, as compared with untreated neurons. The percentage of annexin V-APC stained neurons that were 7AAD negative were considered to be apoptotic (AnnexinV+/7AAD−). (**b**) As revealed by western blot, treatment with 2 μM nutlin-3a for 2 hours under normoxic conditions (indicated as control) increased MDM2, p53, p21 and PUMA expression levels. A representative western blot image is shown out of three. (**c**) Nutlin-3a-induced p53 stabilization did not cause the activation of caspase-3, when compared with neurons treated with the inductor of apoptosis, etoposide (10 μM, 24 hours; in red). (**d**) Disruption of MDM2-p53 interaction induced by nutlin-3a treatment prevented p53 destabilization caused by NMDA-PC. (**e**) Fluorescence microphotographs of neurons after immunostaining for Map-2 (green) revealed that nutlin-3a abrogates NMDA-PC-induced p53 destabilization and promotes neurite degeneration, as judged by quantification of the average primary neurite length and Map-2-staining area, at 4 hours after OGD. Scale bar: 10 μm. (**f**) MDM2-p53 disruption abrogated NMDA-PC-induced neuroprotection at 4 hours after OGD in wt neurons, (**g**) but not in p53 ko neurons. Data are means ± S.E.M. Statistical analysis of the results was evaluated by one-way analysis of variance, followed by the least significant difference multiple range test. Student’s t-test was used for comparisons between two groups of values. In all cases, p < 0.05 was considered significant. In (**a**) *p < 0.05 *versus* untreated neurons. In (**c**,**e**,**f** and **g**) *p < 0.05 *versus* Nx (data not shown in **e**,**f** and **g**) and in all cases ^#^p < 0.05 *versus* OGD. Relative protein abundances quantification Fig. 6b and Fig. 6d are presented in Supplementary Fig. S2 and that “full-length blots/gels are presented in Supplementary Fig. S3.

To investigate the role of the disruption in the interaction between MDM2 and p53 on NMDA-PC-neuroprotection we used a small-molecule nutlin-3a, a potent and specific inhibitor of p53 binding to MDM2. For this purpose, neurons were treated with different concentrations of nutlin-3a (0.1–10 μM) and time periods, as indicated in Fig. 6a. Our results showed a dose-time dependent apoptotic effect of nutlin-3a in treated neurons, as compared with untreated neurons (Fig. 6a). Furthermore, treatment with 2 μM nutlin-3a for 2 hours under normoxic conditions (Nx; indicated as control) increased MDM2, p53, p21 and PUMA expression levels, as revealed by western blot analysis (Fig. 6b). However, nutlin-3a-induced p53 stabilization did not cause the activation of caspase-3, when compared with neurons treated with the inductor of apoptosis, etoposide (10 μM, 24 hours; in red). Indeed, MDM2-p53 disruption induced by nutlin-3a treatment prevented p53 destabilization caused by NMDA-PC (Fig. 6d). Phase contrast microscopy revealed that nutlin-3a abrogated the NMDA-PC-prevented neurite degeneration, as judged by quantification of the average primary neurite length (Fig. 6e) at 4 hours after OGD. The MDM2-p53 disruption abrogated NMDA-PC-induced neuroprotection (Fig. 6f) at 4 hours after OGD in wt neurons but not in p53 ko neurons (Fig. 6g), which corroborate that under NMDA-PC prior ischemia the MDM2-p53 disruption-induced apoptosis is mediated by p53. Furthermore, immunofluorescence analysis showed that nutlin-3a abrogated NMDA-PC-induced p53 destabilization and NMDA-PC-induced inactivation of caspase-3 (see supplementary Fig. S4a and Fig. S5) at 4 hours after OGD.Then, the MDM2-p53 interaction plays a key role in NMDA-PC-caused neuroprotection after ischemia.

### IPC attennuates ischemia-induced infarct growth and increases MDM2 protein levels and p53 destabilization *in vivo*

**Figure 7:**
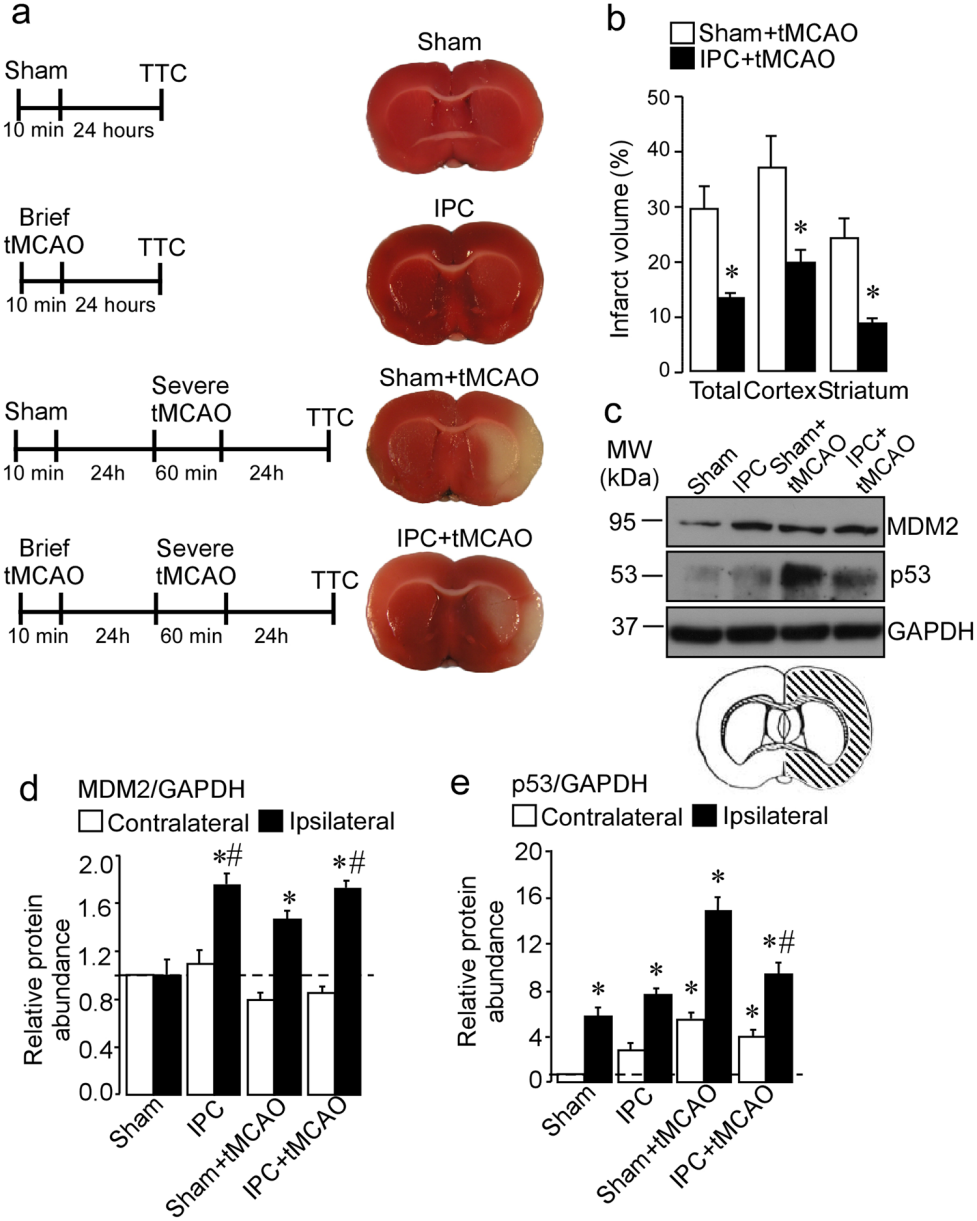
IPC attenuated ischemia-induced infarct growth and increases MDM2 protein levels and p53 destabilization *in vivo*. (a) IPC was generated by 10 min of transient occlusion of the middle cerebral artery previous to tMCAO for 60 min in rat. Sham-operated rats underwent the same surgical procedure except for tMCAO. After 24 hours of tMCAO recovery, rats were euthanized for brain TTC-staining and the results show that (**a**) IPC attenuates tMCAO-induced infarct growth by (**b**) total 60%, cortex 46% and striatum 31% in preconditioned animals (IPC + tMCAO), as compared to non-preconditioned animals prior ischemia insult (SHAM + tMCAO). (**c**) Cortical brain extracts were analyzed by western blotting and (**d**,**e**) the relative abundance of protein levels was quantified after 24 hours of tMCAO recovery. (**c**) IPC increased MDM2 protein levels (**d**) and promoted p53 destabilization (**e**) in the ipsilateral cortex (patterned area) in preconditioned animals (IPC + tMCAO), as compared to non-preconditioned animals previous tMCAO (SHAM + tMCAO). Representative western blot images are shown out of five. GADPH protein levels were used as loading control. Data are means ± S.E.M. (n = 5 independent western blot assays). Statistical analysis of the results was evaluated by one-way analysis of variance, followed by the least significant difference multiple range test. Student’s t-test was used for comparisons between two groups of values. In all cases, p < 0.05 was considered significant. *p < 0.05 *versus* Sham group ^#^p < 0.05 *versus* sham + tMCAO group.

In order to study the *in vivo* relevance of the MDM2-p53 pathway in PC-induced neuroprotection described in cultured cortical neurons, we used a previous validated model of ischemic PC (IPC) and ischemia (tMCAO) in rat^35,36^. IPC was generated by 10 min of transient occlusion of the middle cerebral artery (IPC) prior to tMCAO occlusion for 60 min, as described in material and methods. After 24 hours of tMCAO recovery, we carried out the neurofunctional evaluation, we measured the brain infarct size, and analyzed protein expression in the cortex brain extract by western blot. Our results showed that IPC attenuated brain infarct size (Fig. 7a). As is shown in Fig. 7b, IPC prevented infarct growth by total 60%, cortex 46% and striatum 31% in preconditioned animals (IPC + tMCAO), as compared to non-preconditioned (Sham + tMCAO) animals. Furthermore, Fig. 7c shows that IPC increased MDM2 protein levels but decreased p53 stabilization in the ipsilateral cortex section to infarct zone in preconditioned animals (IPC + tMCAO), compared with not preconditioned animals prior to tMCAO (Sham + tMCAO), as revealed by relative protein abundances quantification (Fig. 7d,e). Finally, median neurofunctional score was 3 (Q_1_ = 3 − Q_3_ = 3.75) in IPC + tMCAO animals and 4 (Q_1_ = 3 − Q_3_ = 4.5) in Sham + tMCAO animals, thus showing a tendency to improve neurofunctional outcome in preconditioned animals (p = 0.1896; using Mann Whitney test). Then, we confirmed in an IPC *in vivo* model that IPC increases MDM2 protein levels and promotes p53 destabilization prior to lethal ischemia, which may contribute to IPC-induced neuroprotection against an ischemic insult.

## Discussion

Here we show the novel relevance of the MDM2-p53 signaling pathway in PC-induced neuroprotection against a subsequent ischemic in both cultured cortical neurons (*in vitro*) and in rat brain (*in vivo*).

We corroborate previous studies showing that conditioning prior to a subsequent ischemic insult, prevents ischemia-induced neuronal apoptosis^9–11^, however the mechanism of PC-mediated neuroprotection is still unknown. Our previous results corroborated that apoptosis is activated after brain ischemia and the its inhibition determines the good prognosis of patients with stroke^32^. In heart, previous studies have revealed that p53 as playing a role in IPC^37^. We previously described that p53 mediates ischemia-induced neuronal apoptosis^32^. Accordingly, here we found that OGD caused p53 stabilization and genetic deletion of p53 totally prevented neuronal apoptosis caused by ischemia. Moreover, we describe that NMDA-PC prevents ischemia-induced p53 stabilization in neurons, which may be related to the neuroprotective role of NMDA-PC against an ischemic insult. Thus, our results reveal that NMDA-PC prevents ischemia-induced p53 stabilization, abrogating ischemia-induced p53-mediated neuronal apoptosis. Here we study the effect of NMDA-PC in the ischemia-induced pathway activation.

In aggreement with several studies showing that PUMA controls caspase-3 activation associated to ischemia in cardiomyocytes and tumor cells^38,39^, we found that NMDA-PC prevents ischemia-induced activation of the p53/PUMA/caspase-3 signaling pathway and promotes neuroprotection against a subsequent ischemic damage. In addition, we show that, whereas p53 mRNA levels remained unaltered, OGD promoted p53 phosphorylation and stabilization, which were prevented by NMDA-PC. These results indicate that NMDA-PC prevents ischemia-induced p53 stabilization throught a posttranslational mechanism (phosphorylation). Indeed, the decrease in p53 phosphorylation caused by NMDA-PC may mediate p53 destabilization and neuroprotection exerted by NMDA-PC prior to the ischemic insult. However, further studies would be necessary for this conclusion. In this context, several kinases are activated in response to ischemia, which promotes p53 phosphorylation of key region-binding sites for MDM2^23,26^ and causes modifications in both p53 and MDM2 proteins leading to a decreased in the avidity of p53 for MDM2^23^. Furthermore, p53 suffer a phosphorylation cascade that first requires phosphorylation of p53 S15, which inhibits its binding to MDM2 and promotes subsequent p53 stabilization in cancer cells^40^. Our results reveal that NMDA-PC increased MDM2 protein levels and promotes p53-destabilization after ischemia, indicating that NMDA-PC-neuroprotection may be regulated by the MDM2-p53 pathway. Our results show that NMDA-PC prevents the OGD-induced p53 nuclear and cytosolic stabilization induced at 4 hours of reoxygenation after ischemia. Indeed, the NMDA-PC-increases MDM2 protein levels in nucleus and cytosol, which facilitates the subsequent p53 destabilization after ischemia. Moreover, our results demonstrate that NMDA-PC increases MDM2 levels and promotes MDM2-p53 interaction, which may lead p53 nuclear and cytosolic stabilization and finally prevents p53-mediated apoptosis after ischemia. These results confirm the relevance of MDM2 in the control of p53 stabilization associated to neuronal ischemia.

Although ischemia increases MDM2-p53 interaction, however this effect is not enough to prevent p53 stabilization, p53/MDM2 relative protein abundance and the subsequent activation of the apoptotic pathway. These results are in agreement with the existence of the auto-regulatory feedback loop between p53 and MDM2, which acts as a repressor mechanism of cellular p53 activity^24,27,30^. Thus, p53 stimulates the expression of MDM2, which, in turn, promotes p53 degradation^27,31^. Our results reveal that NMDA-PC promotes deregulation between the balance of MDM2 and p53, which allows neuronal survival in response to ischemic damage. Moreover, our results show that NMDA-PC-increased direct protein-protein interaction between MDM2 and p53, which is essential to control the proteins levels and activity of p53-pathway induced by ischemia. Furthermore, our results support the existence of other compatible mechanisms with MDM2-induced p53 destabilization responsible for NMDA-PC-caused neuroprotection.

In good agreement with these results, we show that pharmacological blockade of MDM2-p53 interaction with nutlin-3a treatment^41–44^, abrogates NMDA-PC-mediated neuroprotection against an ischemia insult. Then, the nutlin-3a-induced MDM2-p53 disruption promotes a dose and time-dependent induction of apoptosis. Moreover, nutlin-3a binds to MDM2 and inhibits its interaction with p53, which blocks NMDA-PC-mediated p53 destabilization against an ischemic insult. Furthermore, a subtoxic doses of nutlin-3a (2 μM) abrogated NMDA-PC-induced p53-destabilization and promotes neurite degeneration and caspase-3 activation after ischemia. Alltogether our results support a beneficial impact of increased MDM2 protein levels induced by NMDA-PC, which promotes MDM2-p53 interaction and prevents ischemia-induced p53 stabilization. Indeed, this mechanism may be essential for NMDA-PC-mediated neuroprotection. Then, our data reveal MDM2 as a potential therapeutic target associated to ischemic tolerance in neurons.

Finally, we validated the implication of the MDM2-p53 pathway in IPC-induced neuroprotection using a rat brain IPC model (*in vivo)*. Thus, our results show that IPC attenuates ischemia-induced infarct growth, increases MDM2 protein levels and decreases p53 stabilization after cerebral ischemia.

Our present study is focused on early brain preconditioning, where has been described that the most relevant mechanism is the control of the survival/death to prevent the ischemic damage^9–11^. Here, our results corroborated that early PC provides neuroprotection by preventing the neuronal death (necrosis and apoptosis) induced by ischemia. Nevertheless, others mechanisms of delay preconditioning involve in ischemic tolerance, have been described and are compatibles with our results, including those mechanisms that involve inflammation or astrocytic activation^45^. In this context, it has been reported that the MDM2-p53 pathway is involved in ischemia-reperfusion injury and post-conditioning in cultured spinal cord neurons^46^. Future studies would be needed to fully understand the impact of the MDM2-p53 pathway on neuroprotection associated to conditioning prior or after a subsequent brain ischemic insult.

In conclusion, here we show that PC prevents ischemia-induced damage in both cultured neurons *in vitro* and in rat brain *in vivo*. Our results demonstrate that early NMDA-PC confers neuroprotection against ischemia by increasing MDM2 protein level, which promotes its interaction with p53 and triggers p53 nuclear and cytosolic destabilization and prevents ischemia-induced p53-mediated neruronal apoptotic death. Moreover, NMDA-PC attenuated ischemia-induced activation of the p53/PUMA/caspase-3 signaling pathway (see Supplementary Fig. S6). Furthermore, the pharmacological disruption of MDM2-p53 interaction induced by nutlin-3a abrogates NMDA-PC-induced neuroprotection against ischemia. Our results reveal that IPC increases MDM2 protein levels, promotes p53 destabilization and attenuates ischemia-induced infarct growth *in vivo*, with a tendency to improve neurofunctional outcome. These findings demonstrate the key role of the MDM2-p53 pathway in PC-induced neuroprotection against a subsequent ischemic insult and poses MDM2 as an essential target in ischemic tolerance.

## Methods

### Primary cultures of cortical neurons

Neuronal cultures were prepared embryos 14.5E from C57/Bl6/J gestant mice cortices or obtained from *wild type* (wt) or p53-null mice (Tp53^−/−^, knock-out (ko) The Jackson Laboratory; B6.129S2). The colonies were maintained at Animal Experimentation Service of University of Salamanca (USAL) in accordance with Spanish Legislation (RD53/2013). Procedures and Protocols have been approved by the research Bioethics Committee of the USAL. Neurons were seeded at 1.8 × 10^5^ cells/cm^2^ in Neurobasal medium supplemented with 2% B27 and glutamine 2 mM (Invitrogen, Madrid, Spain) and incubated at 37 °C in a humidified 5% CO_2_-containing atmosphere.

### Oxygen glucose deprivation and preconditioning models

After 9–10 DIV, neurons were exposed to oxygen and glucose deprivation (OGD) induced by incubating cells at 37 °C for 90 min in an incubator equipped with an air lock and continuously gassed with 95% N_2_/5% CO_2_. The incubation medium (buffered Hanks’ solution lacked glucose: 5.26 mM KCl, 0.43 mM KH2PO4, 132.4 mM NaCl, 4.09 mM NaHCO3, 0.33 mM Na_2_HPO_4_, 2 mM CaCl_2_, and 20 mM HEPES, pH 7.4) and was previously gassed with 95% N_2_/5% CO_2_ for 30 min. Under these conditions, oxygen concentrations in the incubation medium were 6.7 ± 0.5 lM as measured with a Clark-type oxygen electrode^47,48^. When indicated the neurons were treated with a moderate subtoxic NMDA (20 μM) for 2 hours prior OGD (preconditioning condition, NMDA-PC + OGD). In parallel neurons were incubated in Normoxia (Nx) or preconditioning (NMDA-PC) such as it is described in Table 1.

### Flow Cytometric detection of apoptotic cell death

Neurons were carefully detached from the plates using 1 mM EDTA tetrasodium salt in PBS (pH 7.4) and were stained with annexin V-APC and 7-AAD performed exactly as previously described^49^.

### Trypan blue-staining cells

Necrosis was asseses by the examination of trypan blue-staining cells as previously described^50,51^. Briefly, 4 h of reoxygenation after ischemia, neuronal cultures were washed with warm (37 °C) phosphate-buffered saline (PBS; 136 mM NaCl, 2,7 mM KCl, 7,8 mM Na2HPO4, 1,7 mM KH2PO4, pH 7,4) and incubated with 0,2% trypan blue in PBS for 2 min at room temperature. Microphotographs (20× magnification; Leica) were taken for each experimental condition and viable plus necrotic (stained) cells were counted). At least two different cell cultures utilizing six separate wells were employed, such that a minimum of 7,000–9,000 neurons were counted for each data point.

### LDH release

Neuronal cell injury was quantitatively assessed by the measurement of lactate dehydrogenase (LDH), released from damage or destroyed cells, in the extracellular medium 4 hour after the four conditions studied (Nx, NMDA-PC, OGD and NMDA-PC + OGD). An aliquot of bathing media was combined with NADH and pyruvate solutions^52^. LDH is proportional to the rate of pyruvate loss, which was assayed by absorbance change using a Varioskan Flash (Thermo Fisher, Vantaa, Finland) spectrofluorometer. LDH levels were expressed by percentages. Control experiments have shown previously that the efflux of LDH occurring from either necrotic or apoptotic cells is proportional to the number of neurons damaged or destroyed (Koh and Choi, 1987).

### Quantitative reverse transcription-polymerase chain reaction (RT-qPCR) analysis

Total RNA samples were harvested and purified from culture neurons (GenElute mammalian Total RNA Miniprep kit; Sigma). RT-qPCR was performed using the Power SYBR Green RNA-to-CT TM 1-Step kit (Applied Biosystems, Township, USA). Reverse transcription was accomplished through 30 min at 48 °C cycle, and PCR conditions were 10 min at 95 °C followed by 40 cycles of 15 s at 95 °C plus 1 min at 60 °C. The following forward and reserve primers used were respectively (Thermo Scientific, Offenbach, Germany), 5′-ATTCTGCCCACCACACAGCGACA-3′ and 5′AGGGCTTCCTCTGGGCCTTCTA-3′ (p53), 5′-GGGTGTGAACCACGAGAAAT-3′ and 5′ -GACTGTGGTCATGAGCCCTT-3′ (Gapdh). The mRNA abundance of each transcript was normalized to the Gapdh mRNA abundance obtained in the same sample. The relative mRNA levels were calculated using the ΔΔCt method, and were expressed as the fold change between sample and calibrator.

### Caspase-3 activity assay

Caspase-3 activity was assessed in cell lysates and according to manuacturers´ instructions via Fluorimetric Assay kit CASP3F from SIGMA and read at emission wavelength 405 nm. The method is based on the release of the fluorescent 7-amino-4-methylcoumarin (AMC) moiety. The AMC concentration is calculated using a AMC standard.

### Immunoblots and co-immunoprecipitation assay

Neurons and tissues were lysed in buffer containing 1% SDS, 2 mM EDTA, 150 mM NaCl, 12,5 mM Na2HPO4 and 1% Triton X-100, (NP40: 1%NP40, EDTA diK^+^ 5 mM, Tris pH8 20 mM, NaCl 135 mM y 10% glicerol) supplemented with phosphatase inhibitors (1 mM Na_3_VO_4_ and 50 mM NaF) and protease inhibitors (100 μM phenylmethylsulfonyl fluoride, 50 μg/ml anti-papain, 50 μg/ml pepstatin, 50 μg/ml amastatin, 50 μg/ml leupeptin, 50 μg/ml bestatin and 50 μg/ml soybean trypsin inhibitor), stored on ice for 30 min and boiled for 5 min. Aliquots of lysed extracts were subjected to SDS polyacrylamide gel (MiniProtean®, Bio-Rad) and blotted with antibodies overnight at 4 °C. Antibodies used were anti-p53 (554157, BD Biosciences), anti-pp53 (Ser15; 9286, Cell Signaling, Danvers Massachusetts, USA and anti-cleaved caspase-3 (Asp175, 9661, Cell Signaling), anti-p21(556431, BD Biosciences), anti-MDM2 (2A10, ab-16895), anti-PUMA (ab54288) (Abcam, Cambridge, UK), anti-lamin B (sc-374015, Santa Cruz Biotechnology, Heidelberg, Germany) and anti-GAPDH (Ambion, Cambridge, UK) overnight at 4 °C. After incubation with horseradish peroxidase-conjugated goat anti-rabbit IgG (Pierce, Thermo Scientific) or goat anti- mouse IgG (Bio-Rad), membranes were immediately incubated with enhanced chemiluminescence SuperSignal West Dura (Pierce) for 5 min before exposure to Kodak XAR-5 film for 1 to 5 min, and the autoradiograms were scanned. Band intensities were quantified using ImageJ software^53^.

For co-immunoprecipitation assay, neurons were lysed in ice-cold buffer containing 50 mM Tris (pH 7.5), 150 mM NaCl, 2 mM EDTA, 1% NP-40, supplemented with phosphatase inhibitors described above. After clearing debris by centrifugation, neuronal lysates (100 μg) were incubated with 1 μg of antibody for 24 hours at 4 °C followed by the addition of 10 μl of protein A-agarose (GE Healthcare Life Sciences) for 2 hours at 4 °C. Immunoprecipitates were extensively washed with lysis buffer and before being resolved by SDS-PAGE and immunoblotted with indicated antibodies. The relative protein abundances is shown in Supplementary Fig. S2. Full blots and gel scans are included in Supplementary Fig. S3.

### Subcellular fractionation

To fractionate nucleus from cytosol, neurons were washed with cold PBS containing 1 mM MgCl2, harvested with cytosolic buffer (10 mM HEPES, 1.5 mM MgCl2, 10 mM KCl, 1 mM EDTA, 0.1% NP-40, v/v, 1.5 M sucrose, and protease and phosphatase inhibitors mixture, pH 7.9), triturated with a micropipette to promote cell lysis, left on ice for 30 min, and vortexed for 10 s. After checking cell lysis under a light microscope, extracts were centrifuged at 830 × g for 10 min and the cytosolic fraction (supernatant) was removed and boiled for 5 min. Lysis of the nuclei was performed by resuspending the nuclear pellet in nuclear buffer (50 mM HEPES, 1.5 mM MgCl2, 10 mM KCl mM, 0.5 mM NaCl, 1 mM EDTA, 1% NP-40, v/v, and protease and phosphatase inhibitor mixture, pH 7.9), triturated with a micropipette, left on ice for 2 hours, vortexed (10 s), boiled (5 min), and sonicated (5 min).

### Immunocytochemistry

Neurons grown on glass coverslips were fixed with 4% (v/v, in PBS) paraformaldehyde for 30 min and immunostained with rabbit anti-cleaved caspase-3 (Asp175) (1:300; Cell Signaling Techn, Inc.), mouse anti-MDM2 (2A10, ab-16895), mouse anti-Map2 (SIGMA) antibodies^49^, mouse anti-p53 (554157, BD Biosciences) and rabbit anti-MDM2 (1:500; ab38618 Abcam). Immunolabeling was detected using anti-rabbit IgG–Cy3 or anti-mouse IgG–Cy2 (1:500; Jackson ImmunoResearch; Newmarket, Suffolk, UK). Coverslips were washed, mounted in SlowFade light antifade reagent (Invitrogen) on glass slides, and examined using a microscope (Nikon Inverted microscope Eclipse Ti-E) equipped with 20× objective and a pre-centred fibre illuminator Nikon Intensilight C-HGFI and black and white charge-coupled device digital camera Hamamatsu ORCAER, or on a scanning laser confocal microscope (“Spinning Disk” Roper Scientific Olympus IX81) with three lasers 405, 491 y 561 nm and equipped with 40× and 63× PL Apo oil-immersion objective for high resolution imaging and device digital camera Evolve Photometrics. The Quantification of the average neurite length (Map2 staining) per neuron was performed using the plugin NeuronJ 1.4.0 (ImageJ, version 1.48 v; National Institutes of Health, USA). Values are mean ± SEM from 60 neurons per group measured in three different neuronal cultures. With NeuronJ, the user utilizes a computer mouse to select a starting point and an ending point. NeuronJ makes use of a minimal cumulative cost search algorithm to determine a path between the two points. The algorithm is highly resilient against varying levels of noise and neurite intensity contrast^54^. The degeneration of neurites in the culture was assayed by analyzing the density of Map2-positive neurites in each four groups. Fluorescence 8-bit images were acquired as z stacks using an HCX Plan Apo CS2 63× oil objective and an inverted confocal microscope. Images were exported into ImageJ (1.48 v; National Institutes of Health, USA) in tiff format for processing. Before image analysis, a maximum-intensity projection over z-series projections spanning 2 to 4 μm was performed. Images were converted to grayscale 8-bit images and brightness/contrast was adjusted using the ImageJ “auto” function. All Map2-positive dendrites were automatically delineated using the “auto setting threshold” (default method) and “dark background” functions of ImageJ. Thresholded images were subsequently quantified as percent area (area fraction) using the “analyze-measure” function, which represents the percentage of pixels in the image that have been highlighted (% area). Values are mean ± SEM from 10–15 measurements of 3 different cultures from four different groups^54^. Furthermore, the percentage of neurons with protein-staining is shown in Supplementary Fig. S1.

### Ischemic *in vivo* stroke model: transient middle cerebral artery occlusion (tMCAO)

The animals were anesthetized by i.p. injection of diazepam (5 mg/kg, Almirall Prodesfarma, Barcelona, Spain), ketamine (100 mg/kg, Ketolar, Parke-Davis, El Prat de Llobregat, Barcelona, Spain) and atropine (0.3 mg/kg, B Braun Medical, Rubi, Barcelona, Spain) to perform orotracheal intubation and assisted ventilation (Harvard Apparatus, 683 rodent ventilator, Holliston, MA, USA). Anesthesia was maintained during the procedure with 0.5–1% sevoflurane (Sevorane, Abbott Laboratories, Madrid, Spain). To induce temporary right focal cerebral ischemia, tMCAO was carried out by using the intraluminal thread technique as originally described^36^. Cerebral cortical perfusion (CP) was measured by Laser-Doppler flowmetry together with other physiological parameters^55^. Body temperature was monitored throughout surgery via a rectal probe and maintained at 37 °C by using a heating blanket. After 60 min of ischemia, the intraluminal thread was carefully removed. Sham-operated rats underwent the same surgical procedure except for tMCAO. The surgical incisions were closed, and the rats were allowed to recover from anesthesia, and then placed back into their cages with access to food and water *ad libitum*. Postoperative pain was relieved by s.c. injection of 0.1 mg/kg of buprenorphine (Buprex, Schering-Plough, San Agustín de Guadalix, Madrid, Spain). Twenty-four hours after tMCAO or sham surgery, neurofunctional condition based on four tests, which total score could range from 0 (no neurological deficits) to 6 (highest neurological deficits), was examined just before euthanization^55^. Afterwards, the rats were euthanized under anesthesia and seven 2 mm-thick brain coronal sections were obtained by means of a tissue slicer (Stoelting Wood Dale, IL, USA). Brain infarct size was measured (except for the 3^rd^ coronal section) by the 2,3,5-triphenyltetrazolium chloride (TTC) vital staining method^56^ followed by morphometric analysis with correction for edema^57^. The unstained 3^rd^ coronal sections (0.2 to −1.8 from bregma) were separed into ipsilateral and contralateral hemispheres. The cortex and striatum from each hemisphere were rapidly dissected out, flash-frozen with liquid N_2_ and stored at −80 °C until used for determination of protein expression levels by western blot analysis.

### Ischemic preconditioning (IPC) *in vivo* model

IPC surgery was performed as previously described^35^, with modifications. Briefly, the rats were subjected to 10 min of tMCAO as a preconditioning event followed by a 24 hours period of recovery before the 60 min tMCAO insult. Previous studies have shown that these preceding short ischemic insults produced no brain damage, but did induce tolerance; that is to say, a reduction in the ischemic injury induced by severe tMCAO. Sham rats underwent the same surgical IPC procedure except for tMCAO. Twenty-four hours after IPC or sham surgery, the rats were euthanized for brain TTC-staining and sampling to determine protein expression levels.

### Experimental groups and ethical statement regarding the use of animals

Twenty male Wistar rats (300–350 g, Charles River, Barcelona, Spain) were housed under standard environmental conditions, and fed standard chow with water *ad libitum*. Some rats were excluded from the study according to the following criteria: 1) CP did not drop after filament gliding (no ischemia), n = 1; 2) CP did not recover after filament withdrawal (no reperfusion), n = 1; 3) no brain infarction in spite of a right ischemia-reperfusion pattern, n = 4; and 4) death before the 24 hours time limit, n = 1. Four rat groups were established after exclusions: Sham (n = 2), IPC (n = 2), Sham + tMCAO (n = 5), and IPC + tMCAO (n = 4). Experiments were conducted in compliance with the legislation on protection of animals used for scientific purposes in Spain (RD53/2013) and the EU (2010/63/EU). Protocols were approved by the Animal Experimentation Ethics Committee from IIS Hospital La Fe.

## Electronic supplementary material

Supplementary Figures
