# Supplementary material for: The MDM2-p53 pathway is involved in preconditioning-induced neuronal tolerance to ischemia
Source: Sci Rep. 2018 Jan 25;8:1610. doi: 10.1038/s41598-018-19921-x (PMC5785500; doi:10.1038/s41598-018-19921-x)
Supplement: Supplementary file 1 — Supplementary Figures [file 41598_2018_19921_MOESM1_ESM.pdf]

Supplementary Figures

Title: **The MDM2-p53 pathway is involved in preconditioning-induced neuronal tolerance to ischemia**

Authors: Rebeca Vecino[1,2], Maria C. Burguete[3] , Teresa Jover-Mengual[3], Jesus Agulla[1,4], Veronica Bobo-Jiménez[1,2], Juan B. Salom[3], Angeles Almeida[1,2], and Maria Delgado-Esteban[1,2,*]

[1]Institute of Biomedical Research of Salamanca, University Hospital of Salamanca, University of Salamanca, Calle Zacarías González 2, 37007 Salamanca, Spain
[2]Institute of Functional Biology and Genomics, University of Salamanca, CSIC, Calle Zacarías González 2, 37007 Salamanca Spain
[3]Unidad Mixta de Investigación Cerebrovascular (UMIC), Instituto de Investigación Sanitaria La Fe, and Departamento de Fisiología, Universidad de Valencia, Hospital Universitario y Politécnico La Fe, Av. Fernando Abril Martorell, 106, 46026 Valencia, Spain
[4]Stroke Program. Department of Neurology. Hospital Clínico Universitario, Valladolid. IESCYL Av. Ramón y Cajal, 3, 47003, Valladolid, Spain.

*Corresponding author :
María Delgado-Esteban
Institute of Biomedical Research of Salamanca
University Hospital of Salamanca
University of Salamanca
Calle Zacarías González 2,
37007 Salamanca, Spain
Tel. +34923294908 (5453)
Fax. +34923224876
E-mail: mdesteban@usal.es

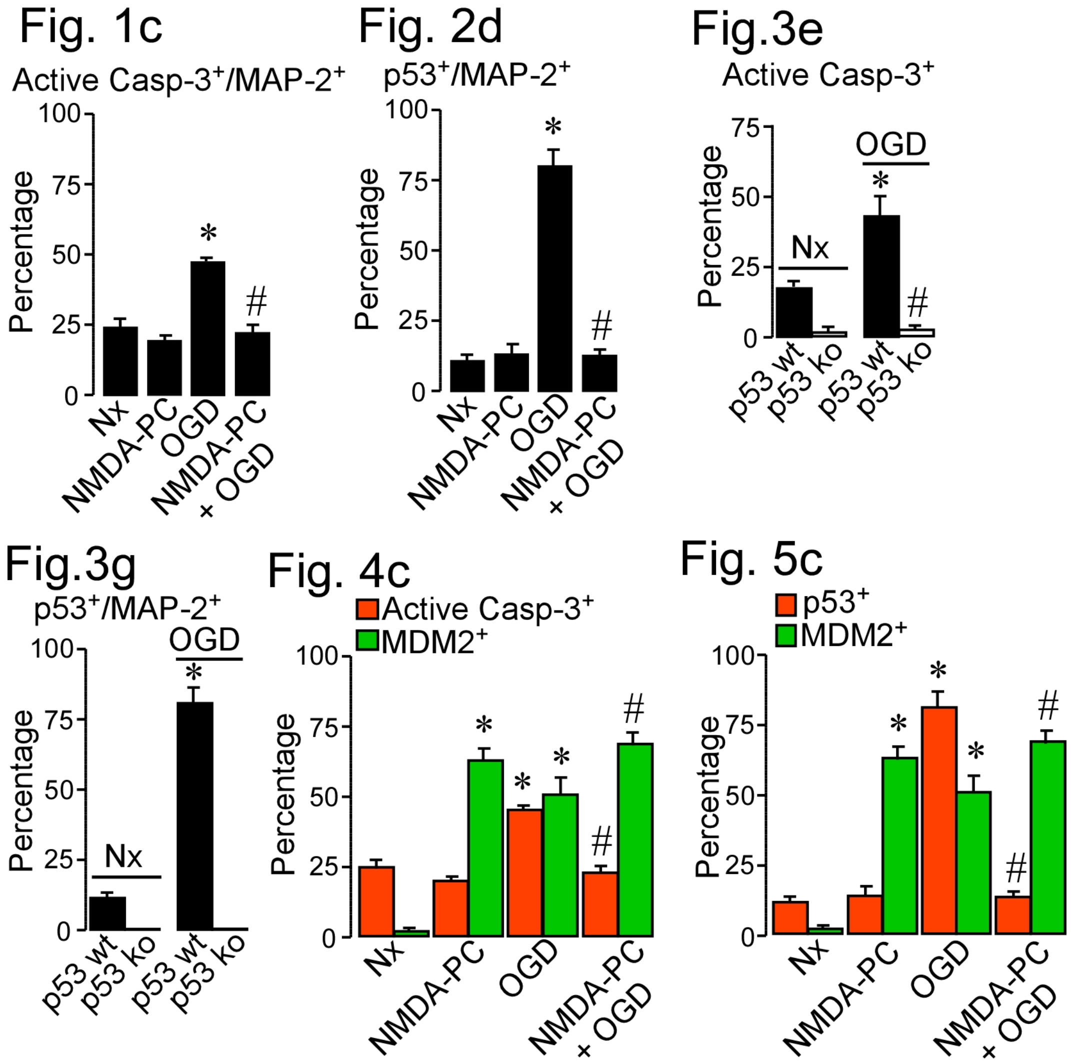

Supplementary Figure S1.Percentage of neurons with protein-staining from results shown in Fig. 1, 2, 3, 4 and 5. Results of the quantification of percentage of active Casp-3^+/Map2^+ neurons of Fig. 1c. Results of the quantification of percentage of p53^+/Map2^+ neurons of Fig. 2d. Results of the quantification of percentage of active Casp-3^+ p53 wt and p53 ko neurons of Fig. 3e and p53^+ of Fig. 3g. Results of the quantification of percentage of active Casp-3^+ and MDM2^+ neurons of Fig. 4c. Results of the quantification of percentage of active p53^+ and MDM2^+ neurons of Fig. 5c. Data are expressed as means ± S.E.M. (n= 3-4 independent experiments). *p<0.05 versus Nx condition. #p<0.05 versus OGD condition.

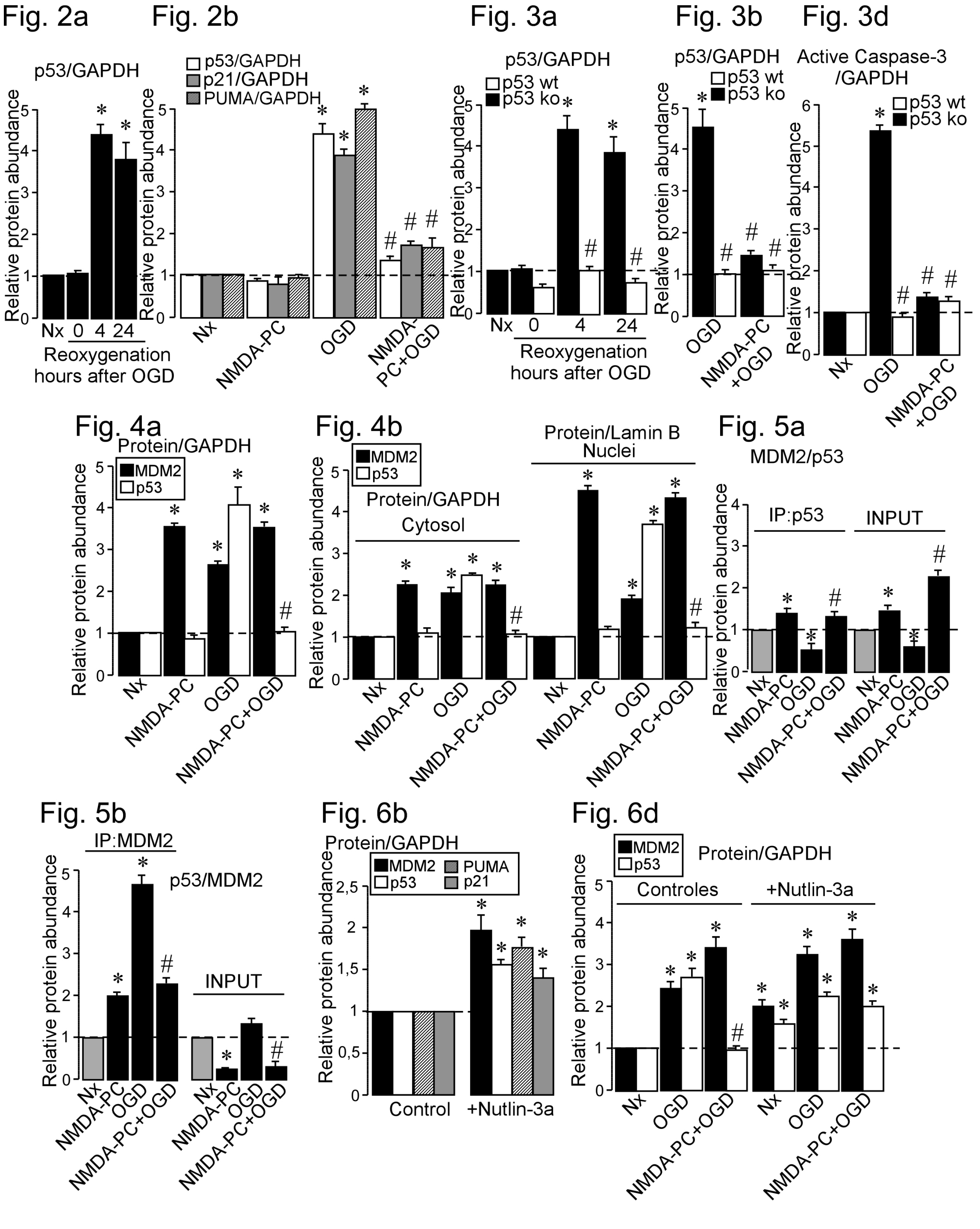

Supplementary Figure S2. Relative protein abundances quantification from results in Fig. 2, 3, 4, 5 and 6. Results of the relative quantification of protein abundance of Fig. 2a, Fig. 2b, Fig. 3a, Fig. 3b, Fig. 3d, Fig. 4a, Fig. 4b, Fig. 5a, Fig. 5b, Fig. 6b and Fig. 6d. Data are expressed as means ± S.E.M. (n=3-4 independent experiments). *p<0.05 versus Nx condition. #p<0.05 versus OGD condition. Band intensities were quantified using the ImageJ software.

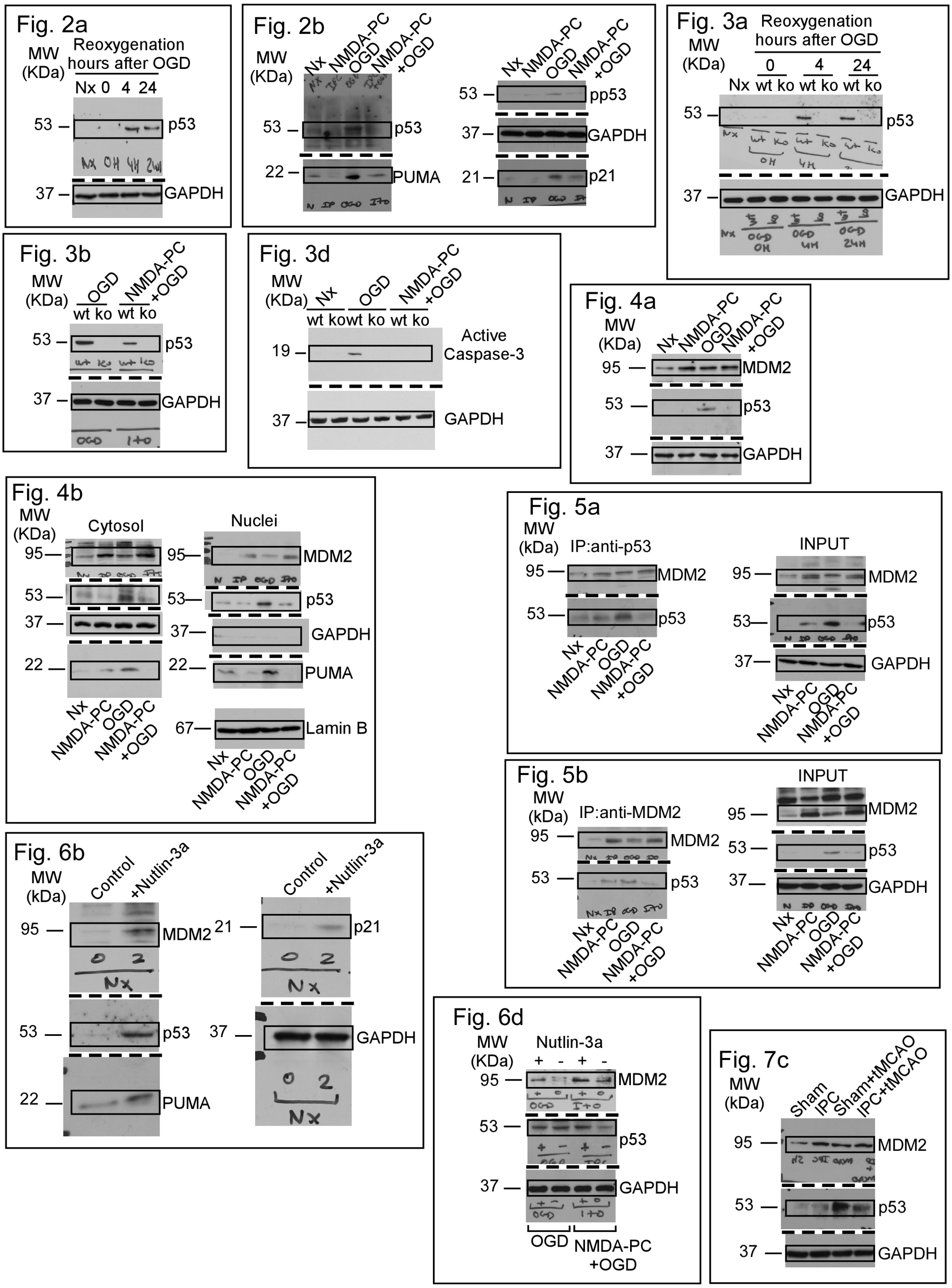

Supplementary Figure S3. Representative full images of immunoblotting. Boxed areas were cropped for designated figures. Dotted lines denote separations of membranes for dual labeling.

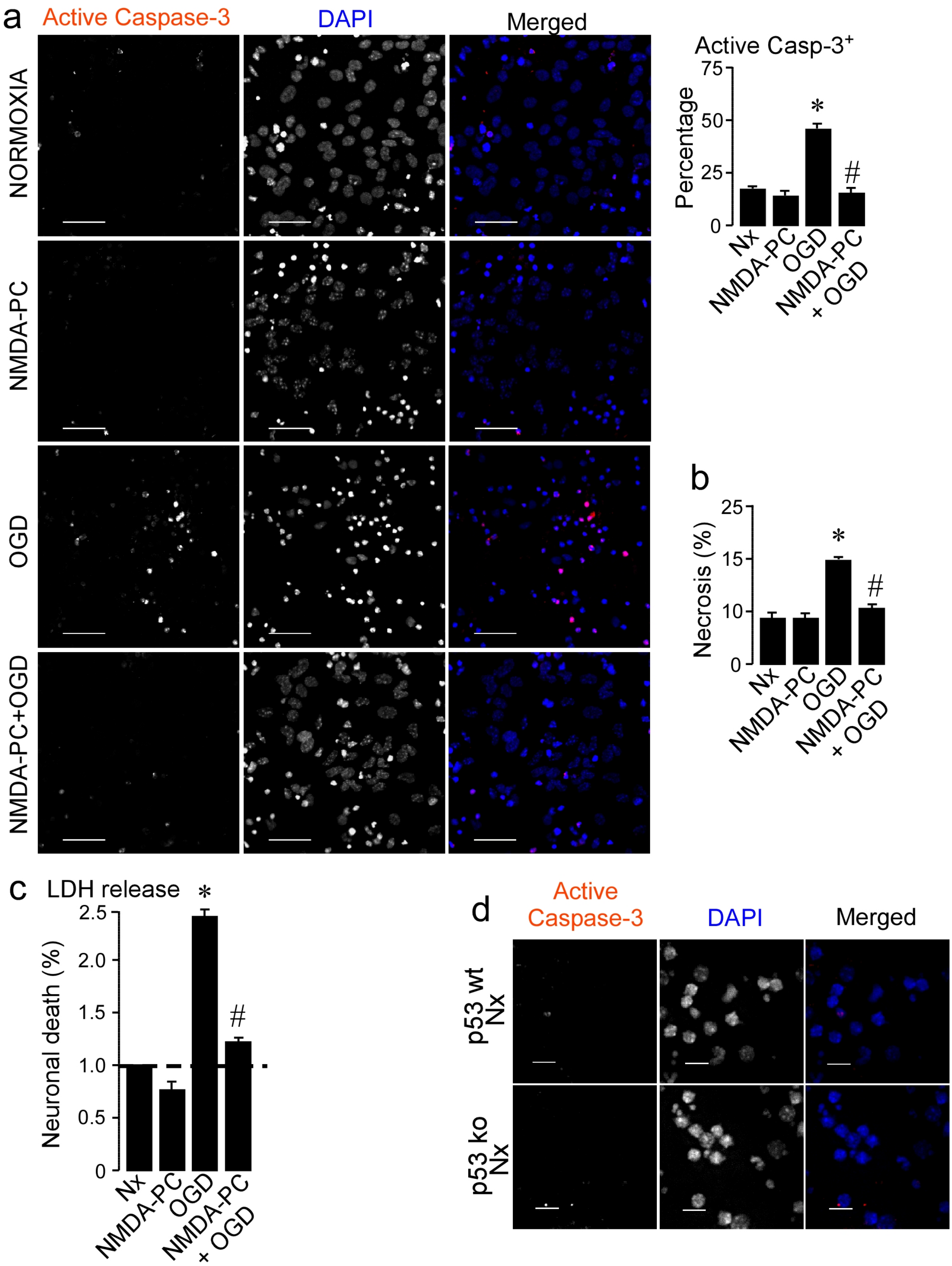

Supplementary Figure S4. NMDA-PC prevents caspase-3 activation, necrosis and neuronal death induced by ischemia. (a) Fluorescence microphotographs after immunostaining for activate Caspase-3 (red) of neurons (9-10 DIV) under Nx, NMDA-PC, OGD and NMDA-PC+OGD and the results of the quantification of percentage of active Caspase-3^{+} neurons. Scale bar 50 μm. (b) Results of quantification of percentage of neuronal necrosis by trypan blue staining and by (c) release of Lactate deshidrogenase (LDH) activity. (d) Fluorescence microphotographs after immunostaining for activate Caspase-3 (red) of wt and ko p53 neurons (9-10 DIV) under Nx (control) condition. Scale bar 20 μm. Data are expressed as means ± S.E.M. (n= 3-4 independent experiments). *p<0.05 versus Nx condition. #p<0.05 versus OGD condition.

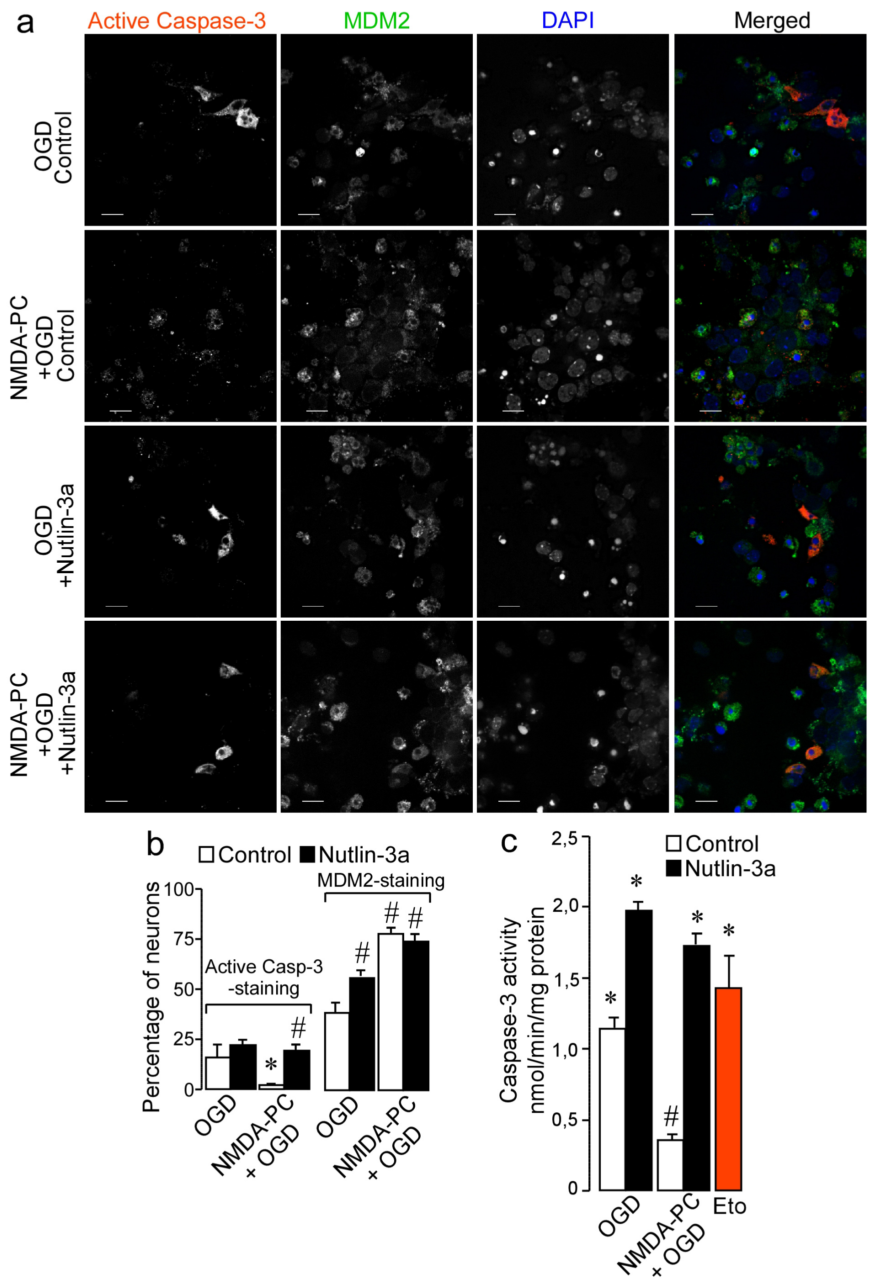

Supplementary Figure S5. Pharmacological disruption of MDM2-p53 interaction abrogates the NMDA-PC-caused Caspase-3 inactivation against ischemia. (a) Fluorescence microphotographs after immunostaining for active Caspase-3 (red) and MDM2 (green) of neurons (9-10 DIV) treated with 2 μM nutlin-3a for 2 hours under Nx, NMDA-PC, OGD and NMDA-PC+OGD. (b) Results of the relative quantification of percentage of neurons with active Casp-3-staining and MDM2-staining. (c) Fluorimetryc asay revealed that the disruption of MDM2-p53 interction abrogated NMDA-PC-prevented caspase-3 activation. Scale bar 20 μm. Data are expressed as means ± S.E.M. (n= 3-4 independent experiments). *$p<0.05$ versus Nx condition. #$p<0.05$ versus OGD condition.

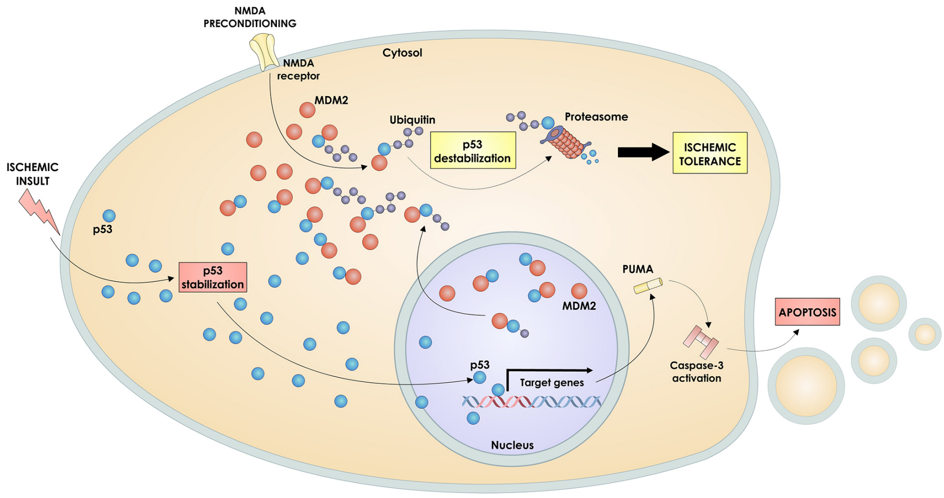

Supplementary Figure S6. Model of NMDA-PC-mediated neuroprotection and ischemic tolerance against ischemia. NMDA-preconditioning confers neuroprotection against ischemia by increasing MDM2 protein levels, which promotes its interaction with p53 and triggers p53 nuclear and cytosolic destabilization and prevents ischemia-induced p53-mediated neuronal apoptotic death. Moreover, NMDA-PC attenuated ischemia-induced activation of the p53/PUMA/caspase-3 signalling pathway. The proposed molecular mechanism reveals the key role of the MDM2-p53 signaling pathway in neuroprotection induced by NMDA-PC against a subsequent ischemic insult and poses MDM2 as an essential target in ischemic tolerance.
